# Influence of phylogenetic, environmental, and behavioral factors on the gut bacterial community structure of dung beetles (Scarabaeidae: Scarabaeinae) in a Neotropical Biosphere Reserve

**DOI:** 10.3389/fmicb.2023.1224601

**Published:** 2023-09-05

**Authors:** Alberto Jácome-Hernández, Araceli Lamelas, Damaris Desgarennes, Carmen Huerta, Magdalena Cruz-Rosales, Mario E. Favila

**Affiliations:** ^1^Red de Ecoetología, Instituto de Ecología A. C., Xalapa, Mexico; ^2^ADM-Biopolis, ADM, Parc Cientific Universitat de Valencia, Paterna, Valencia, Spain; ^3^Red de Biodiversidad y Sistemática, Instituto de Ecología A.C., Xalapa, Mexico

**Keywords:** Coleoptera, gut microbiota, symbionts, metagenomics, 16S

## Abstract

Gut bacteria help dung beetles metabolize nutrients contained and synthesize those unavailable in their food, depending on the ecological scenario in which they develop. However, less is known about the influence of environmental and behavioral factors on the taxonomic composition of bacterial gut communities in Scarabaeinae beetles. To address this research topic, we analyzed 13 tropical dung beetle species in the Los Tuxtlas Biosphere Reserve, Mexico, to understand how the beetle tribe, habitat, food preference, food relocation, and parental care influence the composition of gut bacterial communities. We found that the beetle tribe is the primary factor impacting the taxonomic composition of gut bacterial communities. Among them, Deltochilini displayed the highest variability in diversity due to the different combinations of habitat and food preferences among its species. On the other hand, the other tribes studied did not exhibit such variable combinations. Habitat emerged as the second most influential factor, with forest-dwelling beetles displaying higher diversity. This can be attributed to the heterogeneous environments within tropical forests, which offer a greater diversity of food resources. In contrast, grassland beetles, living in more homogeneous environments and relying on cow feces as their main food source, exhibited lower diversity. Our findings suggest a correlation between bacterial diversity and food resource availability in complex habitats, such as tropical forests, which offer a wider array of food sources compared to simpler environments like grasslands.

## Introduction

The gut bacterial microbiome forms a complex ecosystem known for its major role in the development of its hosts (Hammer et al., 2016; Schwab et al., 2016). It encompasses a myriad of metabolic functions that are absent in the host itself (Engel and Moran, 2013; Estes et al., 2013). In insects, gut bacterial communities (GBC) are involved in various functions, including nutrition, food detoxification, behavior regulation, pathogen control, and even speciation (Douglas et al., 2001; Wernegreen, 2012; Schwab et al., 2016; Boucias et al., 2018).

GBC acquisition in insects depends on a combination of factors, including phylogenetic relationships, dietary needs, habitat, and behavior, among others (Ebert et al., 2021). For instance, termites that cannot degrade lignocellulose acquire intestinal bacteria to perform this crucial task, thus enabling these insects to feed on wood (Bignell et al., 2011). Similarly, the passalid beetle *Odontotaenius disjunctus* relies on its gut bacterial genera *Lactococcus* and *Turicibacter* to degrade wood fibers (Schwarz et al., 2023).

Scarabaeinae beetles, commonly known as dung beetles, are a cosmopolitan insect group comprising more than 8,000 species classified into 12 tribes (Halffter et al., 2013; Tarasov and Dimitrov, 2016; Tonelli, 2021). Dung beetles play a central role in various ecological services, such as organic waste recycling, bioturbation, and controlling noxious groups such as pest insects, parasites, and pathogenic microorganisms. Additionally, they serve as indicators of environmental quality (Nichols et al., 2008). Like other insects, dung beetles depend on their gut bacterial communities to exploit a wide variety of habitats and resources (Estes et al., 2013).

Dung beetles inhabit diverse ecosystems, including tropical forests, where certain species feed on feces from various animals (coprophagous beetles), others scavenge carrion from animal carcasses (necrophagous beetles), and some feed on a combination of feces and decaying fruit (generalist beetles). In grassland fields, dung beetles primarily rely on cow dung as their food source, known to be a nutrient-poor source (Favila, 2001; Estes et al., 2013). These food sources harbor a high diversity of microorganisms, which dung beetles are likely to acquire to aid in the utilization and synthesis of essential nutrients lacking in these feeding resources (Estes et al., 2013; Suárez-Moo et al., 2020).

Scarabaeinae beetles have developed two food relocation strategies to avoid competition with other groups. The first involves burrowing the food, whereby certain beetle species build tunnels beneath the food source and drag food fragments for storage as feeding masses in food chambers. The second strategy is rolling, whereby certain beetle species cut a food fragment, build a ball with it, and cover it with a layer of soil. Then, they roll the ball a few meters away from the food source and bury it slightly for feeding or nesting (Halffter and Matthews, 1966).

Once away from the food source, dung beetles mate, and female burrowers oviposit one egg in a fragment of the food mass, mixing it with soil and their own feces to create a brood mass. Similarly, female rollers add soil and their own feces to the food ball, transforming it into a brood ball where an egg is oviposited (Halffter and Edmonds, 1982). This active behavior is known to be involved in the vertical transmission of gut microorganisms to their offspring, allowing them to efficiently utilize their feeding resources (Parker et al., 2019).

In some beetle species, the female leaves the nest immediately after ovipositing, while in others, the female remains in the nest until the development of their brood is completed and the offspring emerge as adults. This latter behavior is known as parental care (Halffter et al., 2013) and may be related to the control of pathogenic fungi in the nest by their gut microbiota (Favila, 1993; Kim et al., 2021).

Recent studies have demonstrated the significant impact of artificial microbiota deprivation in food sources and beetles during the early life stages of *Digitonthophagus gazella* (previously named *Onthophagus gazella*, belonging to the Onthophagini tribe). This deprivation led to delayed larval development, stunted growth, and high mortality under stressful conditions (Estes et al., 2013; Schwab et al., 2016). In *Copris incertus* (Coprini), the taxonomical composition of gut bacterial microbiota changes throughout the development stages due to their feeding changes in each stage (Suárez-Moo et al., 2020). In Australian dung beetles, the main factors influencing the composition of their gut microbiota are hindgut morphology, diet type, and phylogenetic relationships (Ebert et al., 2021).

Despite these insights, less is known about how taxonomical relationships, environmental preferences, and behavior of dung beetles may be related to the taxonomical composition and function of their gut bacterial communities (GBC). In this study, we explored the influence of tribe, habitat, food preferences, food relocation, and parental care on the GBC of female beetles in 13 Scarabaeinae species in the Los Tuxtlas Biosphere Reserve, Mexico. Our ultimate goal was to gain a deeper understanding of how these factors affect the composition of gut bacterial communities and how these communities assist dung beetles in effectively exploiting a variety of habitats and food sources. Our results will contribute to improving the knowledge of the significance of microbial communities in other insect species and their broader implications for ecosystems.

## Materials and methods

### Beetle specimen collection and preparation

This study focused on female beetles of the following species of the Scarabaeinae subfamily (tribe in parenthesis): *Canthon cyanellus, C. femoralis, C. indigaceus chiapas, C. vazquezae*, and *Deltochilum pseudoparile* (Deltochilini tribe; roller beetles); *Digitonthophagus gazella* (an African species introduced to the United States that has expanded its range to the Americas), *Onthophagus batesi*, and *O. rhinolophus* (Onthophagini); *Coprophanaeus corythus* and *Phanaeus endymion* (Phanaeini); *Dichotomius colonicus* (Dichotomiini); and *Copris laeviceps* and *C. lugubris* (Coprini) (all burrower beetles) (Table 1 and Supplementary Table S1). We analyzed only female specimens because they pass their microbiota to their offspring. Beetles were collected at the Los Tuxtlas Tropical Biology Station, UNAM (18°35′01” N, 95°04′25” W; 150 m a.sl.) using pitfall traps baited with isolated human or cow feces. To prevent microbial contamination, we took precautions to avoid direct contact between beetles and food. Once collected, the beetles were preserved in 70% ethanol until further processing. In the laboratory, beetles were washed in 96% ethanol for 1 min, followed by immersion in PBS+Tween solution for 1 min, and then in 70% ethanol for 1 min. Afterward, they were rinsed three times in 5 ml of distilled water for external cleaning. Specimens were dried in sterile, clean Whatman filter paper and new sterile DNase-free Eppendorf tubes.

**Table 1:** Beetle species used in this study and their respective environmental and behavioral factors.

**Beetle species**	**Tribe**	**Habitat**	**Food preference**	**Food relocation**	**Parental care**
*Canthon cyanellus*	Deltochilini	Forest	Necrophagous	Roller	Present
*Canthon femoralis*	Deltochilini	Forest	Coprophagous	Roller	Absent
*Canthon indigaceus chiapas* H^*^	Deltochilini	Grassland	Coprophagous	Roller	Absent
*Canthon indigaceus chiapas* V^*^	Deltochilini	Grassland	Coprophagous	Roller	Absent
*Canthon vazquezae*	Deltochilini	Forest	Coprophagous	Roller	Absent
*Deltochilum pseudoparile*	Deltochilini	Forest	Necrophagous	Roller	Absent
*Digitonthophagus gazella*	Onthophagini	Grassland	Coprophagous	Burrower	Absent
*Onthophagus batesi*	Onthophagini	Grassland	Coprophagous	Burrower	Absent
*Onthophagus rhinolophus*	Onthophagini	Forest	Generalist	Burrower	Absent
*Coprophanaeus corythus*	Phanaeini	Forest	Necrophagous	Burrower	Absent
*Phanaeus endymion*	Phanaeini	Forest	Coprophagous	Burrower	Absent
*Dichotomius colonicus*	Dichotomiini	Grassland	Coprophagous	Burrower	Absent
*Copris laeviceps*	Coprini	Forest	Coprophagous	Burrower	Present
*Copris lugubris*	Coprini	Grassland	Coprophagous	Burrower	Present

^*^Handled by duplicate and managed as two species due to *Canthon indigaceus chiapas* H being attracted to human feces and *Canthon indigaceus chiapas* V being attracted to cow feces.

### Insect dissection and DNA extraction

The digestive tracts of three female beetles per species were extracted using sterile dissection needles under a Leica EZ4 stereo microscope. DNA extraction was performed separately, following the protocol of Latorre et al. (1986). Briefly, beetle guts were homogenized in a separate Eppendorf tube containing 320 μl of a solution containing 10 mM Tris, 60 mM NaCl, 5% (wt/v) sucrose, and 10 mM EDTA, at pH of 7.8. Next, 400 μl of a freshly mixed solution of 1.25% NaDodSO_4_, 300 Mm Tris, 5% sucrose, 10 mM EDTA, and 0.8% diethyl pyrocarbonate (freshly mixed) at pH of 9 was added. The resulting mixture was then incubated at 65°C for 30 min. After incubation, 120 μl of 3 M sodium acetate was added, and the mixture was kept on ice for 45 min. After 10-min centrifugation, the supernatant was mixed with 1 volume of 2-propanol and left to stand at room temperature for 5 min before another 5-min centrifugation. The supernatant was discarded, and the pellet was re-suspended in 250 μl of distilled water with freshly mixed 0.25% diethyl pyrocarbonate, and then left at room temperature for 30 min. Subsequently, 250 μl of distilled water, 0.1 volume of 3 M sodium acetate, and two volumes of ethanol were added, and the mixture was kept on ice for 10 min. Afterward, the mixture was centrifuged for 5 min and washed with 70% ethanol. Any remaining ethanol was dried in a desiccator for 30 min, and the DNA was dissolved in 10 mM Tris/10 mM EDTA at pH of 8.

### Library construction and amplicon sequencing

A nested PCR was performed using the variable-region V3 primers designed by Klindworth et al. (2013). Each 25-μl PCR reaction consisted of 2 μl of genomic DNA, with a total DNA concentration of 50 ng for amplicon library preparation. The second PCR was conducted using 5 μl of the PCR product (~20 ng/μl), 0.25 μl of each primer, and 12.5 μl of KAPA polymerase (KAPA HiFi HotStart ReadyMix, Kapa Biosystems), with TRIS used instead of water as the buffer. The thermocycling conditions were as follows: initial denaturation at 95°C for 3 min, followed by 25 amplification cycles (95°C for 30 s, 55°C for 30 s, and 72°C for 30 s), and a final extension step at 72°C for 5 min. After amplification, amplicons were purified using the Illumina protocol. A DNA sample was mixed with AMPure XP magnetic beads (18.4 μl for each 23 μl sample) and incubated at room temperature for 5 min. The resulting pellet was washed twice with 80% ethanol; once the ethanol had evaporated, TRIS pH 8.5 was added. The mixture was then incubated at room temperature for 2 min, and the supernatant was transferred to a new, clean tube. The purified amplicons were sequenced using the MiSeq Illumina platform, generating 2 × 300 bp paired-end reads (Gao et al., 2021).

### Bioinformatics analysis

Raw reads were demultiplexed using QIIME 2 v2020.2 (Bolyen et al., 2019). Subsequently, sequences were dereplicated and paired using DADA2 to obtain Amplicon Sequence Variants (ASVs). Chimeric sequences were eliminated using QIIME2 (Gao et al., 2021). ASVs with <5 reads per sample and not present in at least two of the 42 samples were excluded from further analysis. To create a phylogenetic tree of the filtered ASVs, the multiple sequence alignment program MAFFT (Multiple Alignment using Fast Fourier Transform) with the GTR+CAT evolutionary model was used in QIIME2 v2020.2. Taxonomic identification was performed using the RDP method with the 138 SILVA 16S rRNA database in the QIIME2 v2020.2 software (https://docs.qiime2.org/2020.2/data-resources/). Afterward, homologous mitochondria and chloroplast sequences were removed. For taxa that could not be identified at the genus level with the SILVA database, manual BLAST searches were conducted on the NCBI, selecting only bacterial genera with a 99% match. The identified taxa reads were grouped into categories of their respective genus names, while unidentified reads were clustered in a category labeled “Others.” A prevalence filtering step was performed on the resulting bacterial genera database, excluding all bacterial genera that were not present in at least two of the three replicates. Finally, a stacked bar plot was created using the relative read values to visualize the structure of the bacterial communities.

### Alpha diversity analysis

To assess the efficiency of the sequencing depth in estimating the diversity of bacterial communities in each sample, a rarefaction analysis was conducted, normalizing the number of readings at 8,310 iterations. The observed ASVs per sample, as well as the Shannon (species richness), Faith PD (phylogenetic relationship leading to an evolutionary measure of diversity), and Pielou (species evenness) indices, were calculated and analyzed based on beetle species, tribe, habitat, food preference, food relocation, and parental care. The data normality and homoscedasticity were validated with the Shapiro-Wilk and Fligner-Killeen tests, respectively. For scenarios with two variables, the Wilcoxon test was applied, while a generalized linear model (GLM) was used for three or more variables, presented in boxplots with the “ggplot2” package (Wickham, 2016). Due to data overdispersion, a Quasi-Poisson distribution was used. In cases where significant differences were observed, a contrast method was employed over the model using the “stringr” (Wickham, 2019) and “gmodels” (Warnes et al., 2022) packages in R (R Studio Team, 2022).

### Beta diversity analysis

The clustering pattern of the gut bacterial communities in all the dung beetle species analyzed was visualized through a constrained principal component analysis (CPCA) using prevalence-filtered data of weighted and unweighted UNIFRAC distances of ASVs, along with the prevalence-filtered data of weighted and unweighted UNIFRAC distances of bacterial genera found in each gut sample. The influence of different environmental and behavioral factors on the formation of these groups was explored using a PERMANOVA. Both analyses were performed using the “Vegan” package in R (Oksanen et al., 2022).

### Differential abundance of taxonomic traits

Beetle species, tribe, habitat, food preference, food relocation, and parental care (Table 1) were considered factors influencing the structure of beetle gut bacterial communities. To represent the key biological traits associated with each factor (tribe, habitat, food preference, food relocation, and parental care), we conducted a differential abundance analysis using prevalence-filtered data of bacterial genera with the “DeSEQ2” package (Love et al., 2014). A heatmap of the top 50 most abundant traits was constructed using “ggplot2.”

## Results and discussion

### Gut bacterial community composition

We analyzed the gut bacterial microbiota of female beetles of 13 dung beetle species belonging to five different tribes, each with distinct ecological and feeding behaviors (Table 1). An average of 294,062.66 ± 48,154.06 high-quality paired reads were obtained and normalized to a count of 8,310 reads. A total of 1,468 ASVs were identified, with an average of 158.4 ± 93.6 ASVs per sample (for more details, refer to Supplementary Table S1). The rarefaction curves of the “observed ASVs” showed that all samples reached the asymptote, indicating that the sequencing depth was sufficient to represent the true diversity of the gut bacterial microbiota (Supplementary Figure S1).

**Figure 1:**
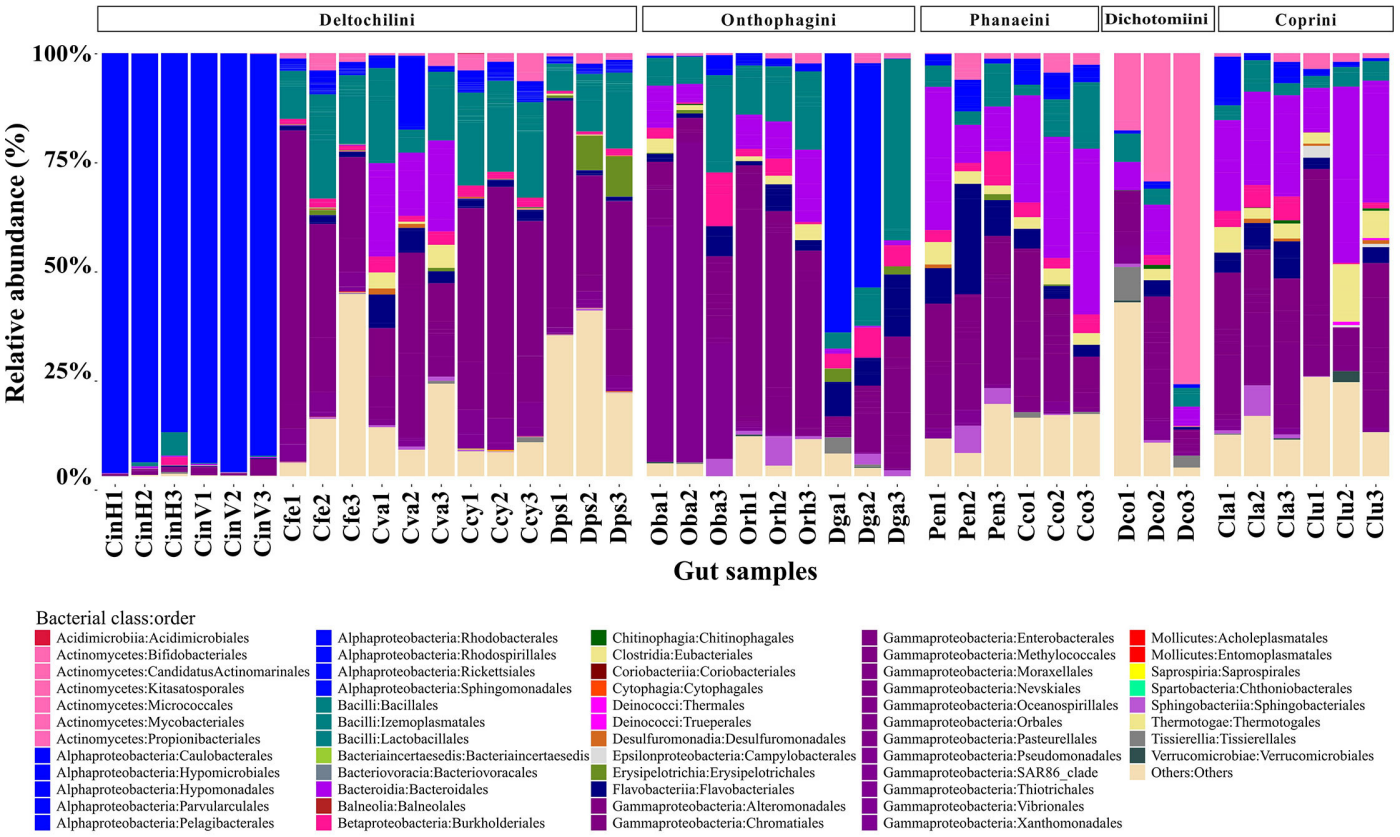
Relative abundance of bacterial class:order per beetle gut sample. Gut bacteria are color-coded by class. Bacteria that were not identified at the genera level collapsed into the “Others” category. Each bar represents a beetle gut sample. Members of the tribe Deltochilini (CinH 1-3: *Canthon indigaceus chiapas* H, CinV 1-3: *Canthon indigaceus chiapas* V, Cfe 1-3: *Canthon femoralis*, Cva 1-3: *Canthon vazquezae*, Ccy 1-2: *Canthon cyanellus*, and Dps 1-3: *Deltochilum pseudoparile*) are on the left, followed by Onthophagini tribe members (Oba 1-3: *Onthophagus batesi*, Orh 1-3: *Onthophagus rhinolophus*, and Dga 1-3: *Digitonthophagus gazella*), Phanaeini tribe members (Pen 1-3: *Phanaeus endymion* and Cco 1-3: *Coprophanaeus corythus*), Dichotomiini tribe members (Dco 1-3: *Dichotomius colonicus*), and Coprini tribe members (Cla 1-3: *Copris laeviceps* and Clu 1-3: *Copris lugubris*).

At the taxonomic level, a total of 229 bacterial genera were identified. The most abundant bacterial classes were Gammaproteobacteria (33.59%), Alphaproteobacteria (19.43%), Bacteroidia (10.22%), and Bacilli (10.19%) (Figure 1). *Wolbachia* (16.64%), *Dickeya* (11.41%) and *Acinetobacter* (5.58%) were the most abundant bacterial genera, with *Acinetobacter* being the only one shared by all beetle species. Notably, the bacterial class Alphaproteobacteria was dominant in *C. indigaceus chiapas* H and V and highly abundant in *Digitonthophagus gazella* due to the bacterial genus *Wolbachia*, which accounted for 95.14% of the gut bacterial community in *C. indigaceus chiapas* H, 96.94% in *C. indigaceus chiapas* V, and 39.5% in *D. gazella*.

**Figure 2:**
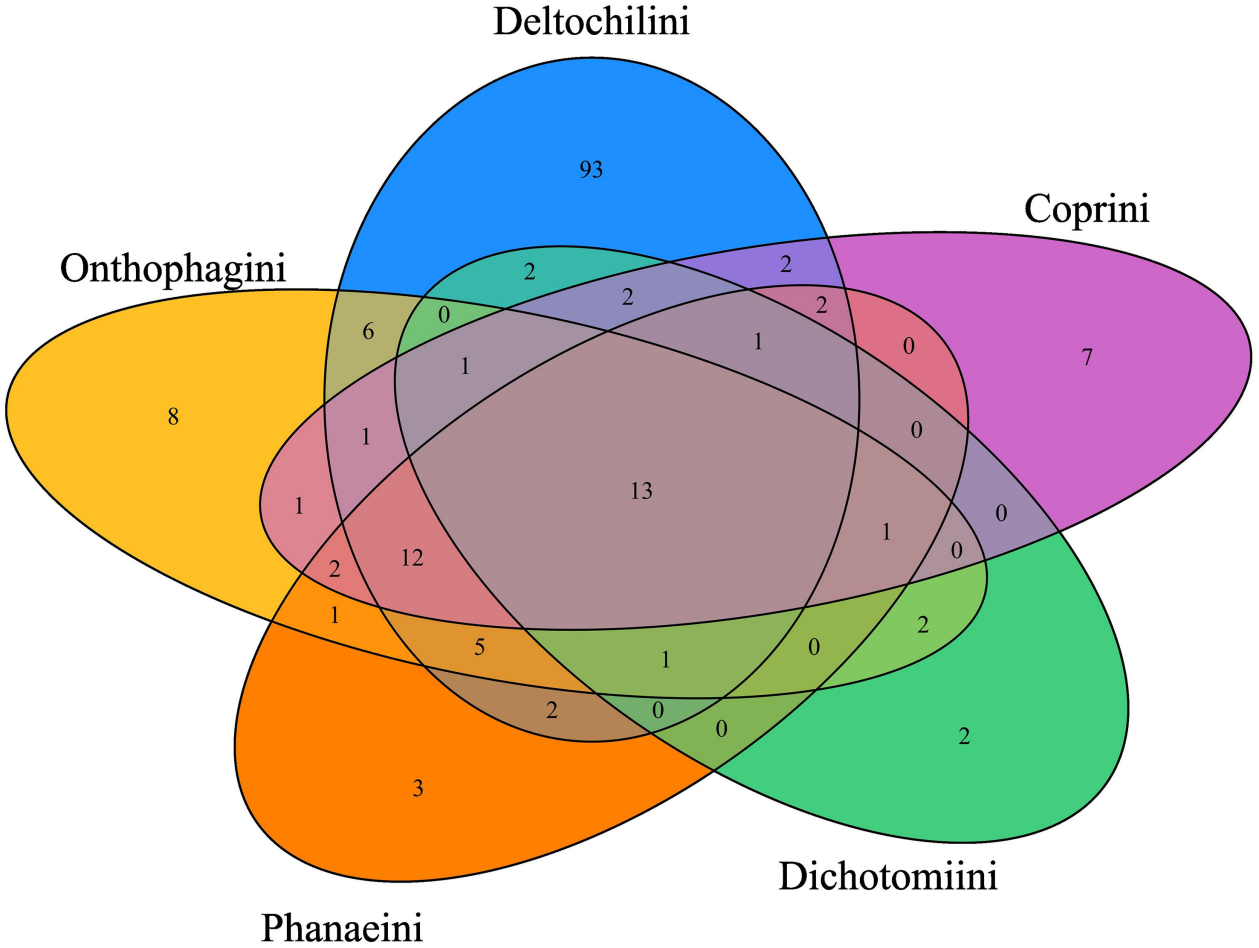
Bacterial genera (13) shared by Scarabaeinae tribes. The Deltochilini tribe has 93 unique bacterial genera, followed by Onthophagini, Coprini, Phanaeini, and Dichotomiini with 8, 7, 3, and 2 unique genera, respectively. Differences between unique bacterial genera in each tribe are the reason why the tribe factor is acting as the main driver in PERMANOVA at ASV and bacterial genera levels.

Regarding the distribution of bacterial genera among Scarabaeinae tribes, 13 bacterial genera (*Acidovorax, Acinetobacter, Bacillus, Bacteroides, Cutibacterium, Duncaniella, Gillisia, Muribaculum, Planococcus, Pseudoalteromonas, Psychrobacter, Sphingobacterium*, and *Vagococcus*) were shared by the five tribes evaluated (Figure 2). However, the number of exclusive bacterial genera varied significantly among the tribes. Deltochilini had 93 unique bacterial genera, while Onthophagini, Phanaeini, Dichotomiini, and Coprini had eight or fewer unique bacterial genera (Figure 2).

### Gut bacterial microbiota diversity

**Figure 3:**
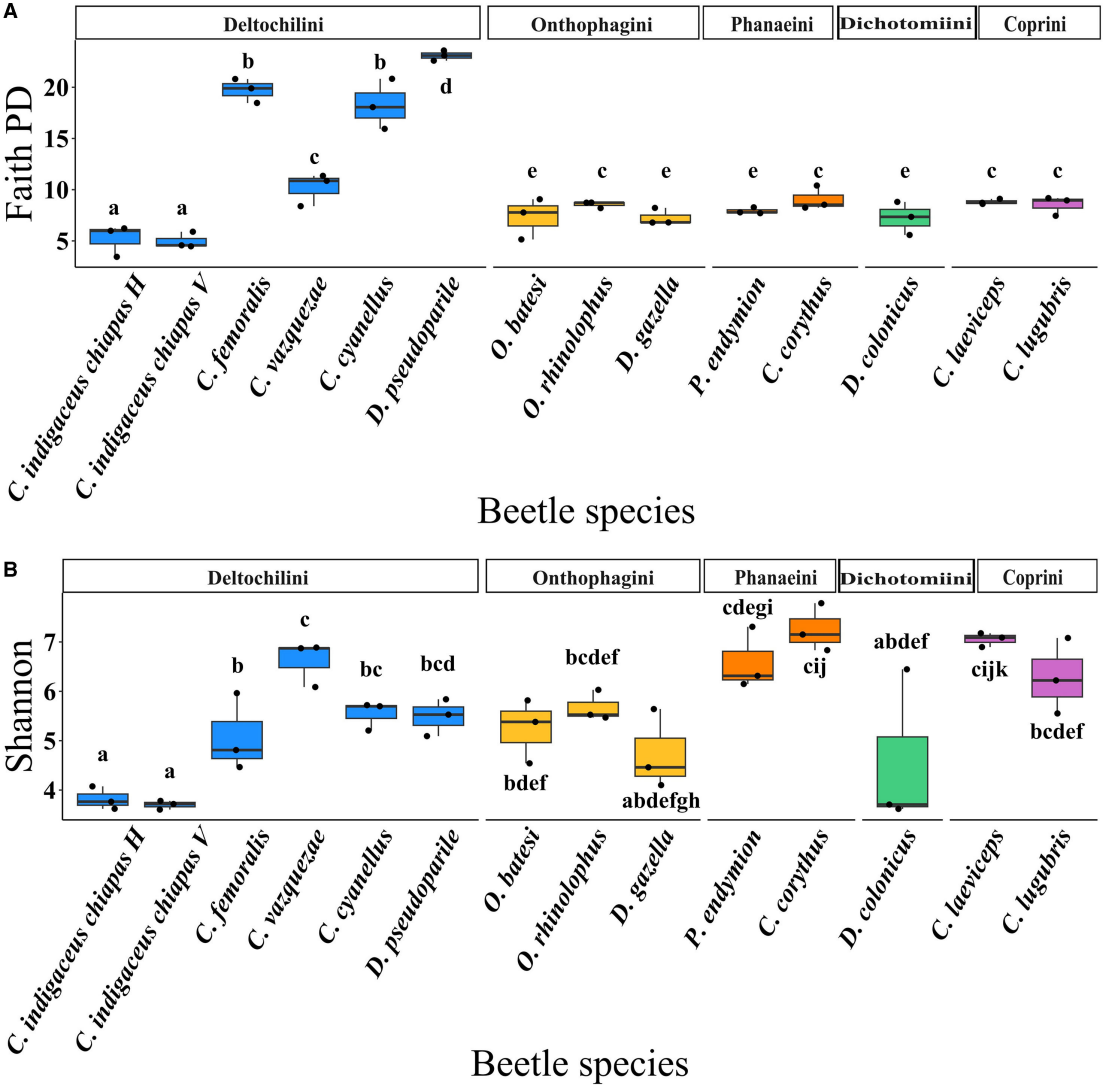
Boxplots showing **(A)** Faith PD index (*t* = 15.387, *p* = 3.44e^−15^) and **(B)** Shannon index (*t* = 15.533, *p* = 2.72e^−15^) in function of beetle species. Means with the same letter are not significantly different.

The gut bacterial diversity within each beetle species was compared based on the observed ASVs (richness) and alpha diversity indices, including Shannon's, Faith PD, and Pielou. When analyzing beetle species (Figure 3), *Canthon cyanellus, C. femoralis*, and *Deltochilum pseudoparile* exhibited the highest observed ASVs (259.33 ± 23.29, 288.66 ± 23.69, and 283 ± 24.83 ASVs, respectively) (Supplementary Tables S2, S3), as well as the highest Faith PD index values (18.27 ± 2.00, 19.72 ± 0.96, and 23.09 ± 0.40, respectively) (Figure 3A and Supplementary Tables S2, S3). These three beetle species live in the forest and belong to the Deltochilini tribe. In contrast, the two variants of *C. indigaceus chiapas* (H and V) exhibited the lowest observed ASVs (46.66 ± 15.75 and 35.66 ± 3.09 ASVs, respectively) (Supplementary Tables S2, S3), the lowest Shannon index values (3.82 ± 0.18 and 3.70 ± 0.07, respectively) (Figure 3B and Supplementary Tables S2, S3), and the lowest Faith PD index values (5.21 ± 1.25 and 4.97 ± 0.64, respectively) (Figure 3A and Supplementary Tables S2, S3). These species also belong to the Deltochilini tribe but inhabit grasslands. Regarding the Pielou index, *C. femoralis, C. indigaceus chiapas* H, and *C. indigaceus chiapas* V had lower values compared to the rest of the species (0.61 ± 0.07, 0.69 ± 0.04, and 0.70 ± 0.01, respectively), indicating an overdominance of some bacterial taxa (Supplementary Tables S2, S3).

In terms of beetle tribe (Supplementary Figure S2 and Supplementary Table S4), the variation in the Deltochilini tribe was due to the presence of both forest and grassland beetle samples, which exhibited high and low observed ASV and indices values, respectively. Based on the habitat factor (Supplementary Figure S3), significant differences were found for observed ASVs (*W* = 33, *p* = 3.72e^−06^), Shannon index (*W* = 76, *p* = 2.15e^−04^), and Faith PD index (*W* = 49, *p* = 5.217e^−06^). In each case, forest beetles showed significantly higher gut bacterial diversity than grassland beetles, while Pielou index values showed no significant difference (*W* = 176, *p* = 0.31).

**Figure 4:**
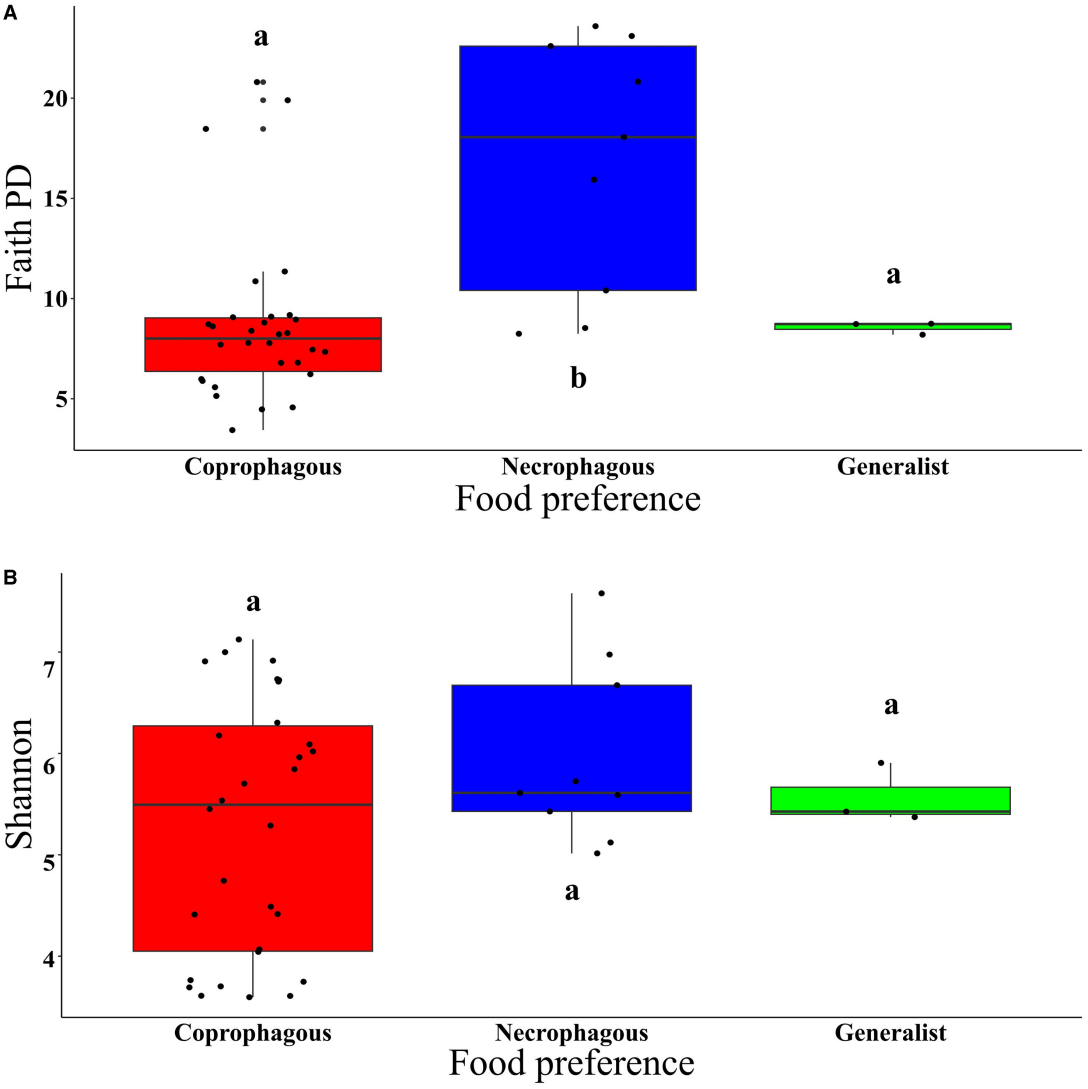
Boxplot of: **(A)** Faith PD index (*t* = 25.037, *p* < 2e^−16^) and **(B)** Shannon index (*t* = 41.115, *p* < 2e^−16^), in the function of beetle food preference. Means with the same letter are not significantly different.

Regarding food preference, coprophagous beetles exhibited significantly higher values for observed ASVs and lower Faith PD index than necrophagous beetles. Generalist beetles did not show significant differences in observed ASVs and the Faith PD index (Figure 4A and Supplementary Table S5). The Shannon and Pielou indices did not show significant differences between coprophagous, necrophagous and generalist beetles (Figure 4B and Supplementary Table S5). Regarding food relocation, burrower beetle samples showed a significantly higher diversity than roller beetles in the Shannon (*W* = 308, *p* = 0.01) and Pielou (*W* = 374, *p* = 2.05e^−05^) indices (Supplementary Figures S4B, D), but not in observed ASVs (*W* = 153, *p* = 0.11) and the Faith PD index (*W* = 149, *p* = 0.09) (Supplementary Figures S4A, C).

The samples of species that exhibit parental care exhibited significantly higher values of the Shannon index (*W* = 81, *p* = 0.03) than samples of species with no parental care (Supplementary Figure S4B). Observed ASVs and the Faith PD and Pielou indices did not show significant differences between the presence and absence of parental care (*W* = 101.5, *p* = 0.15; *W* = 91, *p* = 0.08; and *W* = 103, *p* = 0.17, respectively; Supplementary Figures S5A, C, D).

### Main drivers in the composition of the gut bacterial communities in Scarabaeinae beetles

**Figure 5:**
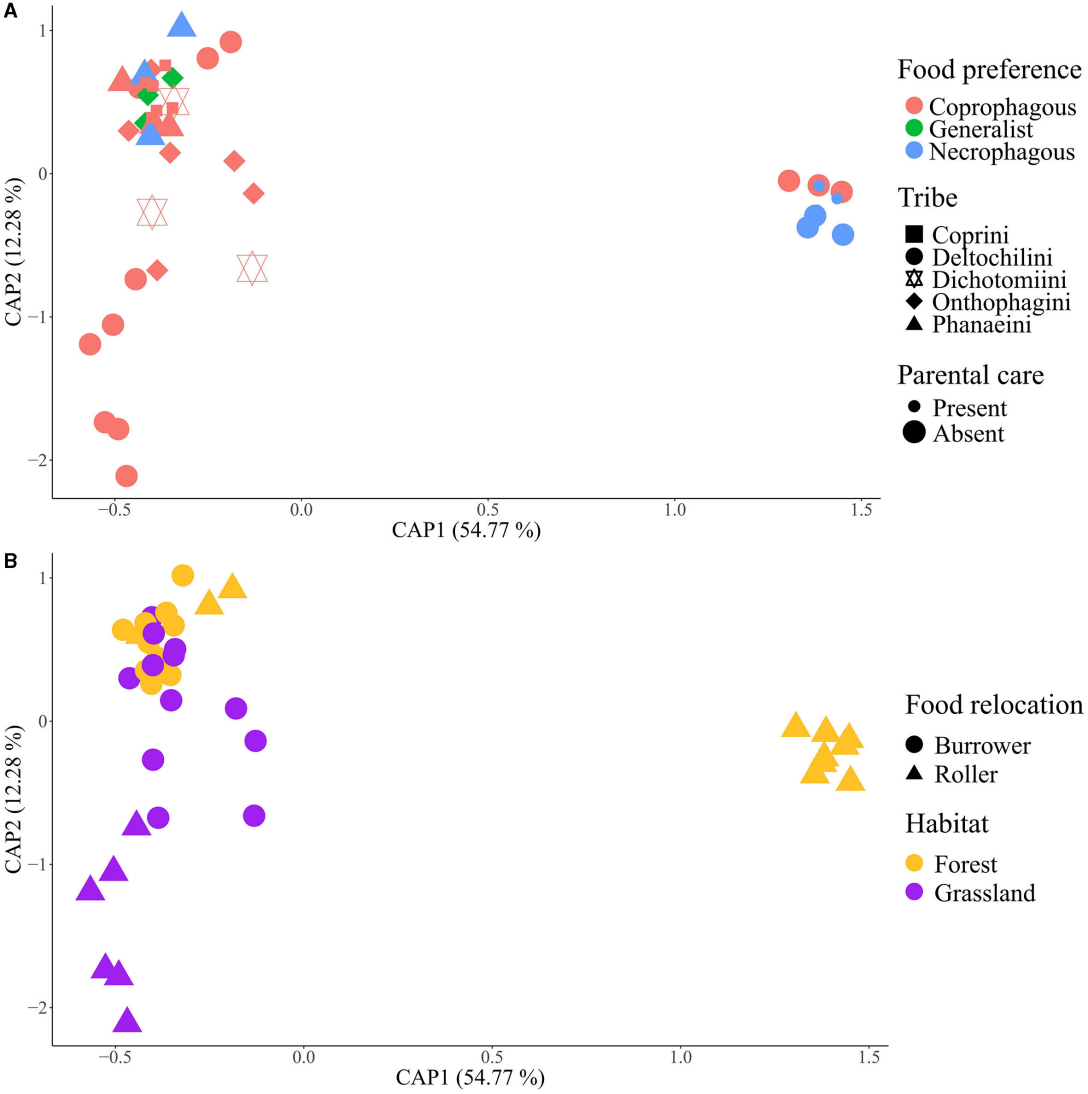
Constrained principal component analysis (CPCA) of intestinal samples based on the ASV unweighted UNIFRAC matrix. **(A)** Tribe, parental care, and food preference factors; **(B)** food relocation and habitat factors. Groups are formed by habitat factor.

The influence of ecological and behavioral factors on gut bacterial community composition was assessed through a constrained principal component analysis (CPCA) using the relative values of weighted and unweighted UNIFRAC of ASVs (Supplementary Figure S6 and Figure 5, respectively). The CPCA revealed distinct clusters among beetle species. *Canthon cyanellus, C. femoralis*, and *Deltochilum pseudoparile* formed a group with high bacterial diversity in their samples. *C. indigaceus chiapas* H and V also formed a distinct cluster, possibly due to the overdominance of the genus *Wolbachia* in their gut bacterial communities, as supported by the similarity with samples from *D. gazella*. The remaining beetle species formed a third cluster with similar gut bacterial communities.

The PERMANOVA results of weighted and unweighted UNIFRAC of ASVs (Supplementary Table S6) indicated the highest covariance with beetle tribe (26.23% and 23.06%, respectively), followed by habitat (15.79% and 10.56%), food preference (6.19% and 10.95%), and parental care (3.42% and 3.26%). Interaction effects were observed between tribe and habitat (5.82% and 4.24%) and between tribe and food preference (2.68% and 3.29%). CPCA and PERMANOVA tests for weighted and unweighted UNIFRAC of bacterial genera showed similar clusters and statistical patterns as the CPCA of ASVs (Supplementary Figures S7, S8 and Supplementary Table S7).

The tribe factor emerged as the main driver of the taxonomical composition of gut bacterial communities in Scarabaeinae beetles for ASV's diversity. Deltochilini, consisting of roller beetles, exhibited the most variable diversity, while the other four tribes (burrower beetles) showed mild variation among them, as evident in the cluster analyses. The variation and imbalance of beetle species in each tribe could explain why the tribe factor is the most important driver of gut taxonomy diversity. Deltochilini had five representative beetle species, Onthophagini had three, Phanaeini and Coprini had two species each, and Dichotomiini had a single species.

To the best of our knowledge, this is the first report of gut microbiota at the bacterial genus level for the Scarabaeinae beetle tribes. However, gut bacterial communities can be highly variable due to beetle diet, mobility capacity, and other factors (Ebert et al., 2021), so further research is needed to determine whether the bacterial cores are persistent.

Habitat is the second most important factor in terms of the taxonomical composition of gut bacteria in terms of ASV's diversity. This may be due to the different functional traits present depending on the habitat where beetles are found. Forest beetles have a higher bacterial species richness compared to grassland beetles, likely because the former live in more heterogeneous environments and have a larger variety of food sources, such as mammal, reptile, and bird feces and carcasses, fruits, and mushrooms (Halffter and Halffter, 1989; Nichols et al., 2008). Thus, they require a high diversity of bacteria to use these different carbon sources. In contrast, grassland beetles inhabit more homogeneous environments and require a smaller diversity of bacteria to use the limited available resources (Estes et al., 2013). Additionally, grassland beetles mainly feed on cow feces, and the low bacterial diversity may be related to the use of antibiotics in cattle (Hammer et al., 2016).

The presence of *Wolbachia* as the overdominant bacterial genus in *C. indigaceus chiapas* H and V and *D. gazella* is intriguing because this bacterial genus is considered an obligate intracellular symbiont in many insect groups, although some endosymbionts can also thrive extracellularly in the midgut (Gosalbes et al., 2010; Pérez-Cobas et al., 2015). Furthermore, some endosymbionts can switch between intra- and extracellular existence, depending on their own needs (Estes et al., 2013). There is also evidence that *Wolbachia* can be part of the gut bacterial communities and may be found in the hindgut of dung beetles of the genus *Onthophagus* (Ebert et al., 2021). Further research is needed to understand the role of *Wolbachia* in the gut bacterial communities of these beetles.

### Abundance of bacterial genera according to the food preferences and habitat of their host

**Figure 6:**
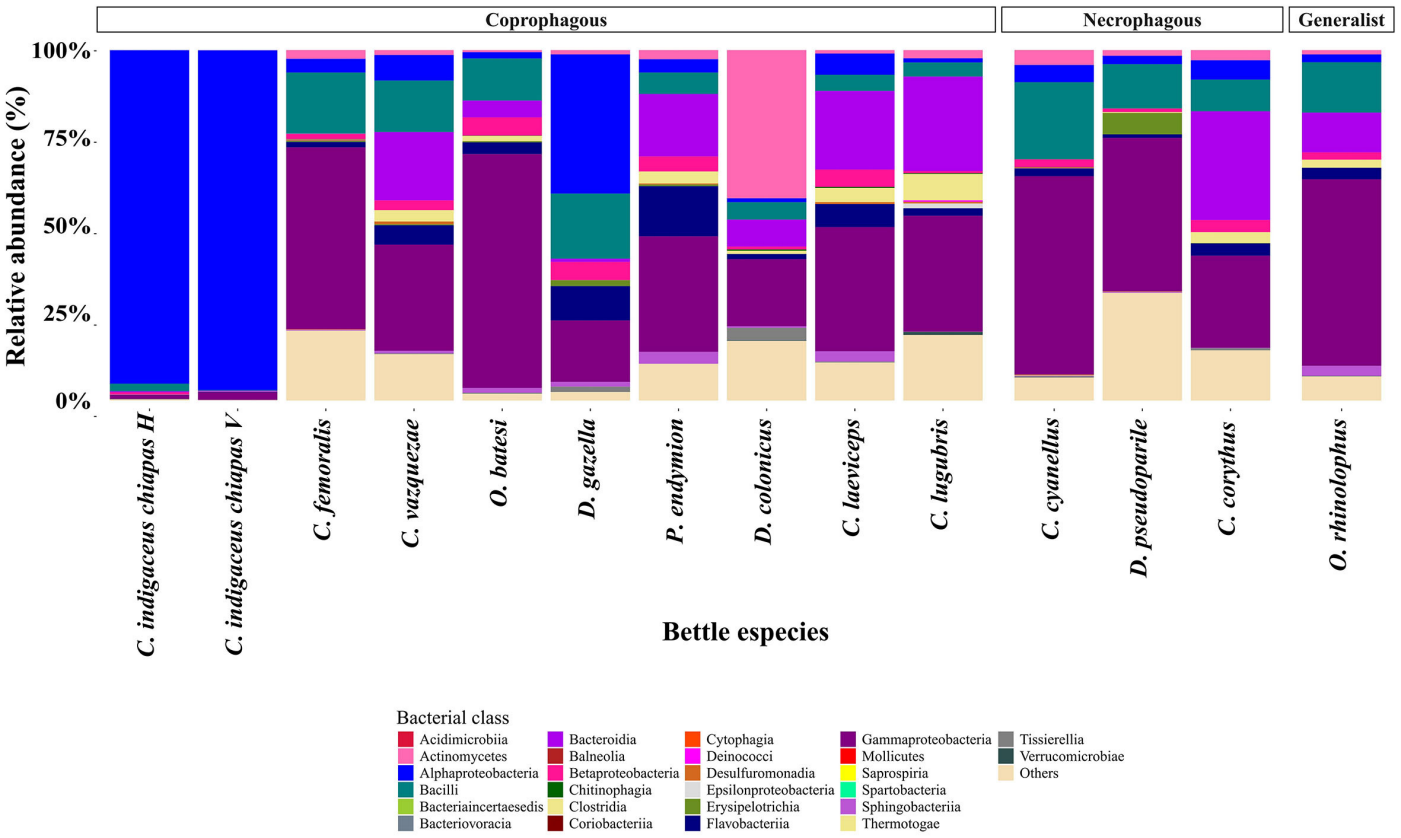
Relative abundance of bacterial classes per beetle species. Gut bacteria are color-coded by class. Bacteria that were not identified at the genera level were collapsed into the “Others” category. Each bar represents a beetle species. Members of coprophagous beetles (*Canthon indigaceus chiapas* H, *Canthon indigaceus chiapas* V, *Canthon femoralis, Canthon vazquezae, Onthophagus batesi, Digitonthophagus gazella, Phanaeus endymion, Dichotomius colonicus, Copris laeviceps*, and *Copris lugubris*) are on the left, followed by necrophagous (*Canthon cyanellus, Deltochilum pseudoparile*, and *Coprophanaeus corythus*) and generalist (*Onthophagus rhinolophus*).

In terms of bacterial class, the structure of bacterial communities varied according to the food preference of each beetle species, consistent with the observed pattern in gut samples (Figure 6).

**Figure 7:**
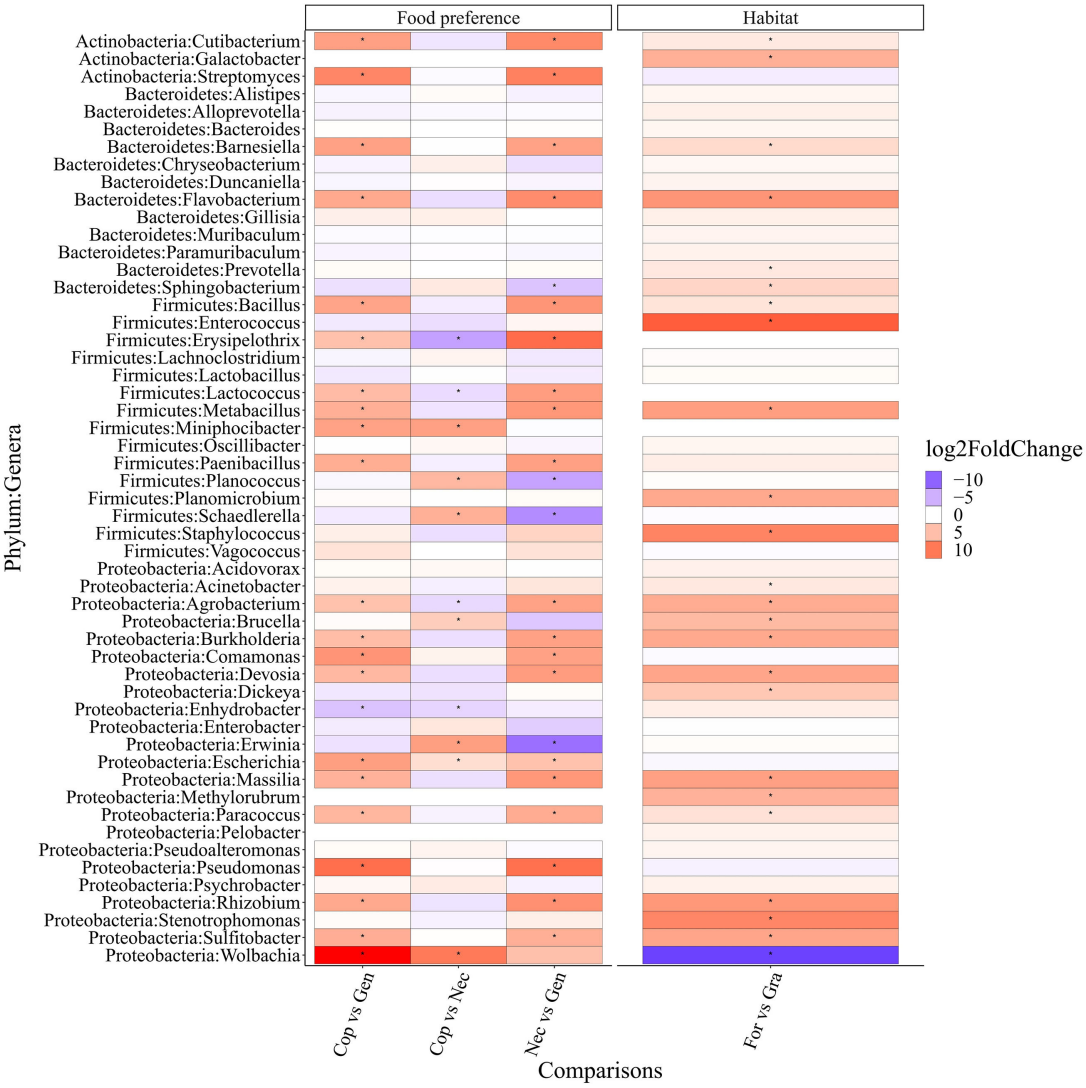
Differential abundance between different conditions is indicated on the x-axis of the top 50 most abundant gut bacterial genera of the Scarabaeinae beetles. Cop, coprophagous; Gen, generalist; Nec, necrophagous; For, forest; Gra, grassland. Dots in the cells indicate significant differences (*p*-value < 0.05).

The differential abundance analysis of the top 50 bacterial genera (Figure 7) revealed significant differences based on food preference. Coprophagous beetles had 28 significantly more abundant bacterial genera, including *Wolbachia, Escherichia, Erwinia, Brucella, Schaedlerella, Planococcus*, and *Miniphocibacter*, compared to necrophagous beetles. Many of these genera are known to produce enzymes that can break down complex carbohydrates from plant cell walls, such as *Erwinia* spp., which can degrade pectin-rich materials (Edwards and Doran-Peterson, 2012). Additionally, the gut of coprophagous beetles contained 21 bacterial genera, including *Wolbachia, Sulfitobacter, Rhizobium, Pseudomonas*, and *Massilia*, that were more abundant than those found in generalist beetles.

On the other hand, necrophagous beetles had 23 significantly more abundant bacterial genera (4 more than coprophagous and 19 more than generalist beetles). Generalist beetles had five significantly more abundant bacterial genera (one more than coprophagous beetles and four more than necrophagous beetles).

When considering habitat preference, forest beetles had 23 significantly more abundant bacterial genera than grassland beetles, which, in turn, had 1 more significantly abundant bacterial genus than forest beetles.

The differential abundance analyses revealed that certain taxonomical traits could act as biomarkers for food preference and habitat conditions in dung beetles. For example, coprophagous beetles had a significantly higher abundance of bacterial genera, such as *Erwinia, Escherichia*, and *Schaedlerella*, known for their ability to degrade lignocellulose (de Souza, 2013). This difference may be attributed to the fact that coprophagous beetles predominantly inhabit grasslands and feed primarily on cattle feces, which contain high amounts of cellulose and lignin (Estes et al., 2013; Parker and Moczek, 2020). Gut bacteria break down these complex compounds and synthesize substances not present in feces, such as certain amino acids.

Another interesting example is the bacterial genus *Lactococcus*, which acts as a biomarker of food preference within the beetle species in this study. *Lactococcus* has been reported as one of the main bacteria responsible for wood-fiber digestion in various insects, including the passalid beetle *Odontotaenius disjunctus*, the wood-feeding cockroach *Panesthia angustipennis*, and some members of the termite group (Schwarz et al., 2023). We found a consistent presence of *Lactococcus* in the gut of beetles feeding on feces with a high amount of plant fibers derived from the diet of herbivorous animals. However, in this study, the abundance of *Lactococcus* was significantly higher in necrophagous beetles compared to coprophagous beetles, in which it was significantly more abundant than in generalist beetles. This abundance pattern may be due to the fact that *Lactococcus* also has great proteolytic capacity, allowing it to participate in multiple functions, depending on the food preference scenario (Li et al., 2020).

Additionally, *Pseudomonas*, known for its significant amino acid metabolism and nitrogen fixation capacity, was more abundant in coprophagous than in necrophagous beetles. These bacteria provide essential amino acids and other nitrogenous compounds that are absent in feces (Parker et al., 2019).

Surprisingly, food relocation did not seem to have a significant impact on the composition of gut bacterial communities in these beetle species, at least in this study. Further research is needed to fully understand the influence of this factor on the gut microbiota of dung beetles.

## Conclusion

Our study indicates that the structure of the gut bacterial microbiota in dung beetles can be significantly influenced by environmental and behavioral factors, in addition to vertical transfer. The presence of certain bacterial genera may be linked to the need for acquiring essential nutrients that are absent in food resources and metabolizing nutrients that are present. However, as gut bacterial communities can be highly variable, we recommend further research not only on the dung beetle species studied in this study but also on additional dung beetle species to gain a comprehensive understanding of the structure and function of the gut bacterial microbiota in the Scarabaeinae group.

## Data availability statement

The datasets presented in this study can be found in online repositories. The names of the repository/repositories and accession number(s) can be found below: https://www.ncbi.nlm.nih.gov/, PRJNA955791.

## Ethics statement

The manuscript presents research on animals that do not require ethical approval for their study.

## Author contributions

AJ-H: study design, bioinformatic analysis, statistical analysis, and draft redaction. DD and MF: study design, statistical advice, and draft review. CH: study design, funding acquisition, and draft review. MC-R: study design and draft review. AL: funding acquisition, study design, sequencing design, bioinformatic analysis, statistical advice, and draft review. All authors contributed to the article and approved the submitted version.
