# Supplementary material for: Influence of phylogenetic, environmental, and behavioral factors on the gut bacterial community structure of dung beetles (Scarabaeidae: Scarabaeinae) in a Neotropical Biosphere Reserve
Source: Front Microbiol. 2023 Sep 5;14:1224601. doi: 10.3389/fmicb.2023.1224601 (PMC10508338; doi:10.3389/fmicb.2023.1224601)

Table S1. Summary of number of reads from the gut microbiota associated to the different Scarabaeinae beetle species analyzed in this study.

Species	Sample ID	Number of reads after quality filtering	Non-chimeric reads	ASV number
Canthon cyanellus	Ccy1	169555	119557	261
Canthon cyanellus	Ccy2	171417	117951	230
Canthon cyanellus	Ccy3	207438	149462	287
Canthon femoralis	Cfe1	339351	273471	269
Canthon femoralis	Cfe2	324063	248730	322
Canthon femoralis	Cfe3	267398	214179	275
Canthon indigaceus chiapas H	CinH1	339015	132115	25
Canthon indigaceus chiapas H	CinH2	358447	151246	62
Canthon indigaceus chiapas H	CinH3	333543	111953	53
Canthon indigaceus chiapas V	CinV1	349953	130592	40
Canthon indigaceus chiapas V	CinV2	338942	158156	33
Canthon indigaceus chiapas V	CinV3	315800	110401	34
Canthon vazquezae	Cva1	281202	65568	180
Canthon vazquezae	Cva2	358516	49539	169
Canthon vazquezae	Cva3	365426	68374	206
Deltochilum pseudoparile	Dps1	309682	244329	248
Deltochilum pseudoparile	Dps2	258789	206757	303
Deltochilum pseudoparile	Dps3	300932	237291	298
Digitonthophagus gazella	Dga1	322547	55183	57
Digitonthophagus gazella	Dga2	311138	88070	127
Digitonthophagus gazella	Dga3	227283	52452	52
Onthophagus batesi	Oba1	273756	78386	198
Onthophagus batesi	Oba2	324871	79579	103
Onthophagus batesi	Oba3	237261	52123	58
Onthophagus rhinolophus	Orh1	258178	62056	136
Onthophagus rhinolophus	Orh2	269082	63935	142
Onthophagus rhinolophus	Orh3	271293	43655	80
Coprophanaeus corythus	Cco1	316168	63068	145
Coprophanaeus corythus	Cco2	248719	92877	263
Coprophanaeus corythus	Cco3	275784	65183	187
Phanaeus endymion	Pen1	295597	48836	211
Phanaeus endymion	Pen2	252644	80950	130
Phanaeus endymion	Pen3	288030	59224	97
Dichotomius colonicus	Dco1	292089	28103	34
Dichotomius colonicus	Dco2	288165	69438	160
Dichotomius colonicus	Dco3	296986	49317	53
Copris laeviceps	Cla1	333960	63545	189
Copris laeviceps	Cla2	369430	93972	160
Copris laeviceps	Cla3	353588	72666	196
Copris lugubris	Clu1	239731	67268	104
Copris lugubris	Clu2	309854	139024	89
Copris lugubris	Clu3	305009	83916	178

Table S2. Number of ASV and Shannon, Faith PD and Pielou indexes of the gut bacterial communities of dung beetle samples.

Sample ID	SV	Shannon	Faith	Pielou
Ccy1	261	5.697224376	18.05723172	0.704368059
Ccy2	230	5.206315577	15.94101752	0.66303508
Ccy3	287	5.719508252	20.82739059	0.698048587
Cfe1	269	4.465240817	18.46330275	0.545311713
Cfe2	322	5.963841312	20.80176926	0.717985129
Cfe3	275	4.810435155	19.89662508	0.586556273
CinH1	25	3.620751452	3.439769156	0.745269586
CinH2	62	3.764948659	6.223176702	0.630439725
CinH3	53	4.075255153	5.982696272	0.708487214
CinV1	40	3.780987617	5.891440832	0.69155122
CinV2	33	3.605300055	4.566366031	0.701412182
CinV3	34	3.717450632	4.472538346	0.721413584
Cva1	180	6.871225445	8.393675179	0.920545363
Cva2	169	6.087183778	10.86116351	0.826824086
Cva3	206	6.886371811	11.34755602	0.902030358
Dps1	248	5.093319717	22.59554533	0.629152751
Dps2	303	5.527751492	23.59187197	0.661013307
Dps3	298	5.838797792	23.0976107	0.702934804
Dga1	57	4.460689394	6.806763886	0.759508329
Dga2	127	5.641017826	8.223312526	0.809783355
Dga3	52	4.099675473	6.791104407	0.725320172
Oba1	198	5.815736124	9.066027713	0.766976241
Oba2	103	4.54137945	7.776246801	0.678633396
Oba3	58	5.381161476	5.140436577	0.918603437
Orh1	136	5.468952964	8.729831473	0.769343049
Orh2	142	6.029683319	8.743636836	0.849880667
Orh3	80	5.526320737	8.200160975	0.874132687
Cco1	145	6.833194264	8.24731267	0.950897521
Cco2	263	7.784477278	10.40669078	0.971750239
Cco3	187	7.150464202	8.530317587	0.947687599
Pen1	211	7.306982333	8.279593097	0.945624865
Pen2	130	6.313426922	7.786581429	0.89989594
Pen3	97	6.148770304	7.707689705	0.931619998
Dco1	34	3.707535424	5.578582386	0.729551324
Dco2	160	6.445929198	8.802908625	0.880368894
Dco3	53	3.61894386	7.345541524	0.635024431
Cla1	189	6.896505652	8.717667882	0.911583022
Cla2	160	7.087344199	8.611662642	0.969448012
Cla3	196	7.175512331	9.099267628	0.941643392
Clu1	104	5.552026307	8.953545688	0.827743426
Clu2	89	6.221006803	7.453636074	0.959160723
Clu3	178	7.079997289	9.176544173	0.947254238

Table S3. GLM of observed ASV and diversity indexes per beetle species

	Observed ASV			Shannon index			Faith PD index			Pielou index		
	**Estimate**	***t*-value**	**Pr (>|*t*|)**	**Estimate**	***t*-value**	**Pr (>|*t*|)**	**Estimate**	***t*-value**	**Pr (>|*t*|)**	**Estimate**	***t*-value**	**Pr (>|*t*|)**
C. indigaceus chiapas H	3.8430	11.671	2.86e^{-12}	1.34033	15.533	2.72e^{-15}	1.6516	15.387	3.44e^{-15}	-0.364229	-7.107	9.85e^{-08}
C. indigaceus chiapas V	-0.2688	-0.537	0.595292	-0.03166	-0.257	0.79874	-0.0468	-0.305	0.762873	0.014377	0.199	0.843635
C. femoralis	1.8222	5.135	1.92e^{-05}	0.28495	2.495	0.01877	1.3301	11.020	1.08e^{-11}	-0.119277	-1.596	0.121707
C. vazquezae	1.3773	3.738	0.000845	0.54899	5.066	2.32e^{-05}	0.6709	5.084	2.2e^{-05}	0.239950	3.503	0.001564
C. cyanellus	1.7151	4.795	4.86e^{-05}	0.37184	3.315	0.00254	1.2540	10.305	4.94e^{-11}	-0.009034	-0.124	0.901905
D. pseudoparile	1.8024	5.072	2.28e^{-05}	0.36198	3.221	0.00323	1.4880	120522	5.42e^{-13}	-0.044692	-0.610	0.546952
O. batesi	0.9417	2.426	0.021976	0.31715	2.796	0.00925	0.3401	2.422	0.022179	0.126062	1.793	0.083728
O. rhinolophus	0.9389	2.418	0.022384	0.39573	3.545	0.00140	0.4953	3.637	0.001101	0.179246	2.581	0.015367
D. gazella	0.5222	1.256	0.219353	0.21439	1.848	0.07515	0.3327	2.365	0.025167	0.096180	1.359	0.185111
P. endymion	1.1406	3.015	0.005408	0.54518	5.027	2.58e^{-05}	0.4184	3.027	0.005251	0.287038	4.233	0.000224
C. corythus	1.4469	3.954	0.000476	0.64150	6.017	1.75e^{-06}	0.5524	4.101	0.000321	0.320045	4.754	5.44e^{-05}
D. colonicus	0.5677	1.377	0.179277	0.18372	1.573	0.12696	0.3284	2.333	0.027081	0.074297	1.044	0.305402
C. laeviceps	1.3591	3.682	0.000980	0.61314	5.723	3.87e^{-06}	0.5242	3.871	0.000593	0.303301	4.489	0.000112
C. lugubris	0.9746	2.522	0.017641	0.49773	4.549	9.51e^{-05}	0.4918	3.609	0.001186	0.271440	3.990	0.000432

Table S4. GLM of observed ASV and diversity indexes per beetle tribe

	Observed ASV			Shannon index			Faith PD index			Pielou index		
	**Estimate**	***t*-value**	**Pr (>|*t*|)**	**Estimate**	***t*-value**	**Pr (>|*t*|)**	**Estimate**	***t*-value**	**Pr (>|*t*|)**	**Estimate**	***t*-value**	**Pr (>|*t*|)**
Deltochilini	5.20979	45.371	$2e^{-16}$	1.61754	36.598	$2e^{-16}$	2.60864	29.109	$2e^{-16}$	-0.34434	-12.877	$3.06e^{-15}$
Onthophagini	-0.54740	-2.258	0.0299	0.03463	0.458	0.649892	-0.56486	-2.965	0.00527	0.11453	2.567	0.0144
Phanaeini	-0.06133	-0.261	0.7956	0.31730	4.023	0.000272	-0.46940	-2.175	0.03606	0.28379	5.879	$9.16e^{-07}$
Dichotomiini	-0.79901	-1.838	0.0742	-0.09348	-0.768	0.447449	-0.62870	-2.004	0.05240	0.05441	0.787	0.4362
Coprini	-0.18153	-0.737	0.4656	0.27989	3.503	0.001220	-0.44892	-2.098	0.04277	0.26761	5.513	$2.87e^{-06}$

Table S5. GLM of observed ASV and diversity indexes per beetle food preference

	Observed ASV			Shannon index			Faith PD index			Pielou index		
	**Estimate**	***t*-value**	**Pr (>|*t*|)**	**Estimate**	***t*-value**	**Pr (>|*t*|)**	**Estimate**	***t*-value**	**Pr (>|*t*|)**	**Estimate**	***t*-value**	**Pr (>|*t*|)**
Coprophagia	4.85826	46.890	$<2e^{-16}$	1.68109	41.115	$<2e^{-16}$	2.16584	25.037	$<2e^{-16}$	-0.22583	-7.842	$1.5e^{-9}$
Necrophagia	0.65068	3.795	0.000503	0.12630	1.557	0.128	0.65617	4.591	4.51e-05	-0.03569	-0.587	0.560
Generalism	-0.07634	-0.215	0.831234	0.05498	0.416	0.680	-0.01899	-0.066	0.948	0.04085	0.436	0.665

Table S6. PERMANOVA results for weighted and unweighted UNIFRAC matrices of intestinal ASV

	Weighted UNIFRAC					Unweighted UNIFRAC				
	df	MS	F	R^2	Pr(>F)	*df*	MS	F	R^2	Pr(>F)
Tribe	4	0.23434	5.1003	0.26230	0.001	4	0.46343	3.9984	0.23067	0.001
Habitat	1	0.56438	12.2832	0.15792	0.001	1	0.84905	7.3255	0.10565	0.001
Food preference	2	0.11063	2.4078	0.06191	0.009	2	0.43996	3.7959	0.10950	0.002
Parental care	1	0.12238	2.6635	0.03424	0.023	1	0.25449	2.1957	0.03167	0.047
Tribe:food preference	1	0.09596	2.0885	0.02685	0.051	1	0.26480	2.2846	0.03295	0.043
Tribe:habitat	1	0.20799	4.5266	0.05820	0.001	1	0.34124	2.9442	0.04246	0.013
Residuals	31	0.04595		0.39857		31	0.11590		0.44710	
Total	41			1.00000		41			1.00000	

Table S7. PERMANOVA results for weighted and unweighted UNIFRAC matrices of intestinal bacterial genera

	Weighted UNIFRAC					Unweighted UNIFRAC				
	df	MS	F	R^2	Pr(>F)	*df*	MS	F	R^2	Pr(>F)
Tribe	4	0.19440	11.6067	0.39646	0.001	4	0.40237	3.7954	0.22906	0.001
Habitat	1	0.41719	24.9087	0.21271	0.001	1	0.80509	7.5942	0.11458	0.001
Food preference	2	0.04079	2.4351	0.04159	0.019	2	0.37393	3.5271	0.10643	0.002
Parental care	1	0.00529	0.3159	0.00270	0.873	1	0.12150	1.1555	0.01743	0.263
Tribe:food preference	1	0.02964	1.7695	0.01511	0.156	1	0.18607	1.7552	0.02648	0.114
Tribe:habitat	1	0.13083	7.8114	0.06671	0.001	1	0.26907	2.5381	0.03829	0.024
Residuals	31	0.01675		0.26473		31	0.10601		0.46772	
Total	41			1.00000		41			1.00000	

Figure S1. Rarefaction curves based on the “Observed ASV” of the intestinal samples of the studied Scarabaeinae beetles.

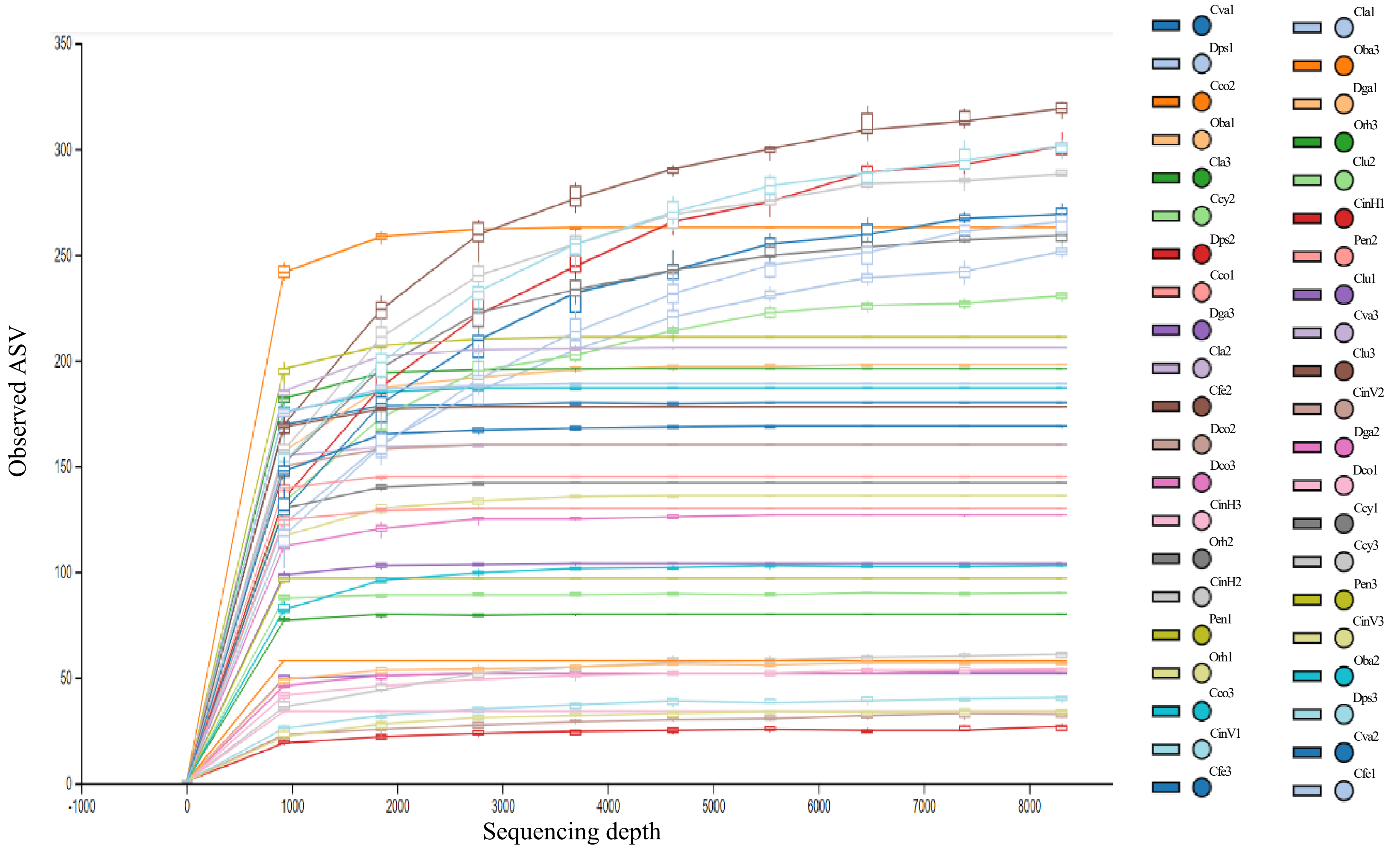

Figure S2. Boxplot of: A) observed ASV, B) Shannon index, C) Faith PD index and D) Pielou index, in function of beetle tribe. Groups are represented by lowercase letters.

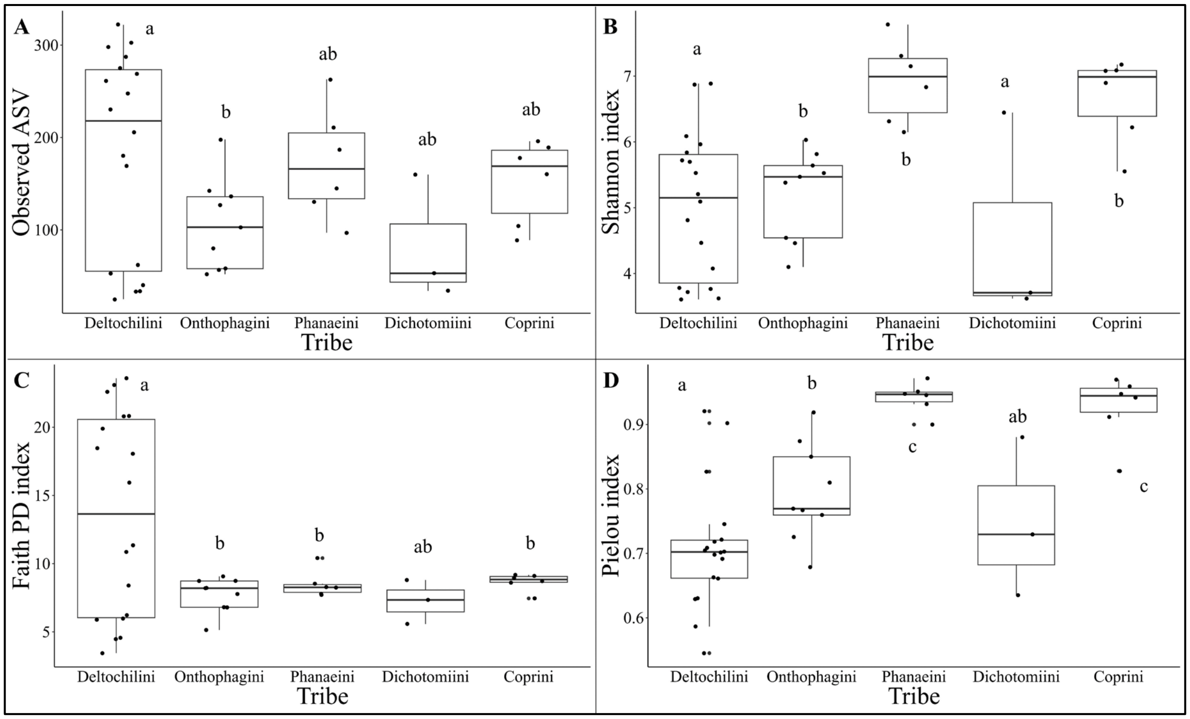

Figure S3. Boxplot of: A) observed ASV, B) Shannon index, C) Faith PD index and D) Pielou index, in function of beetle habitat. Groups are represented by lowercase letters.

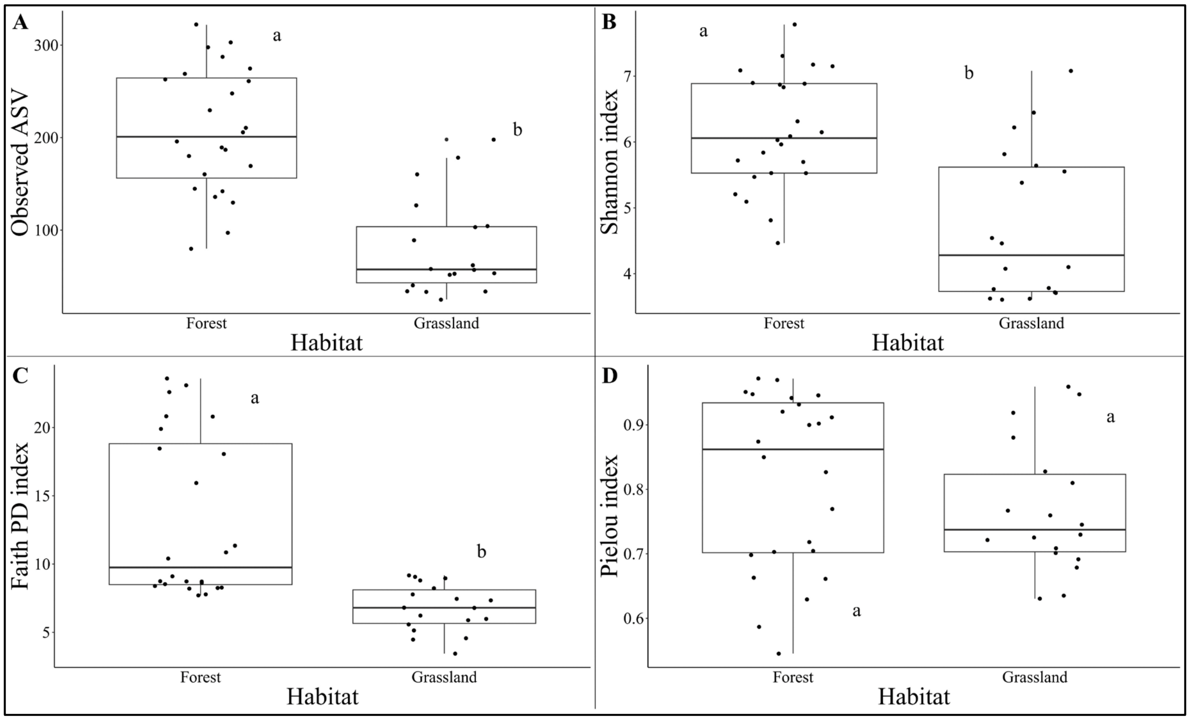

Figure S4. Boxplot of: A) observed ASV, B) Shannon index, C) Faith PD index and D) Pielou index, in function of beetle food relocation. Groups are represented by lowercase letters.

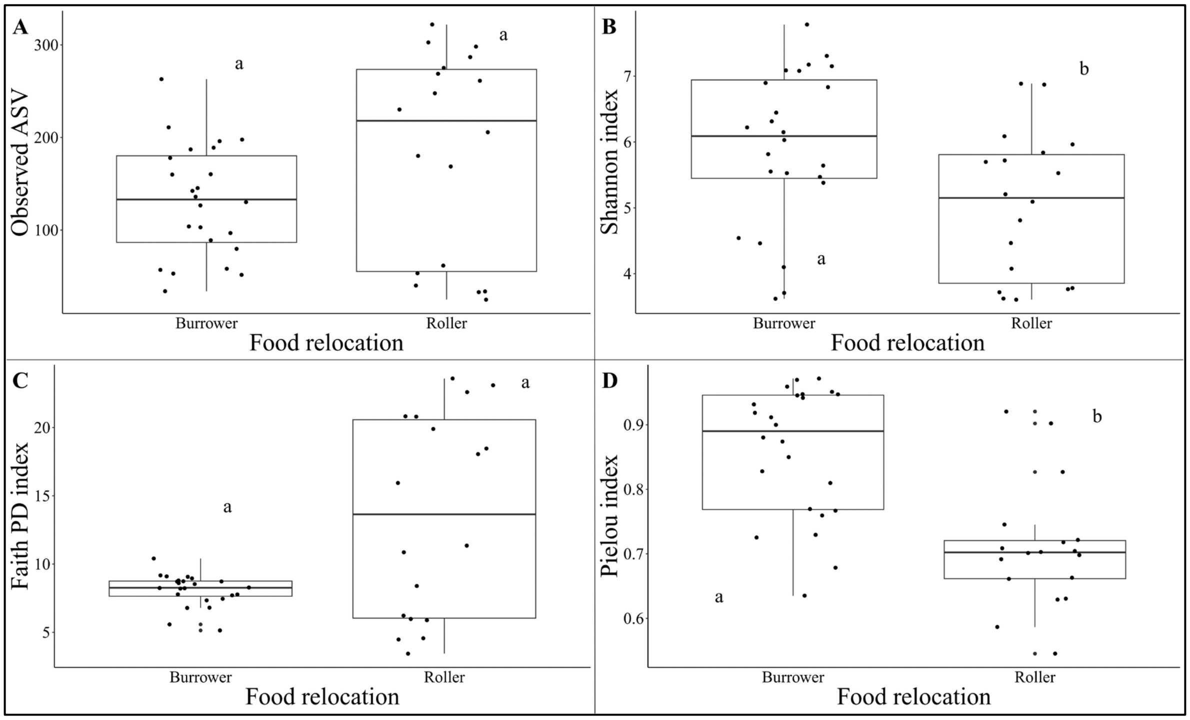

Figure S5. Boxplot of: A) observed ASV, B) Shannon index, C) Faith PD index and D) Pielou index, in function of beetle parental care. Groups are represented by lowercase letters.

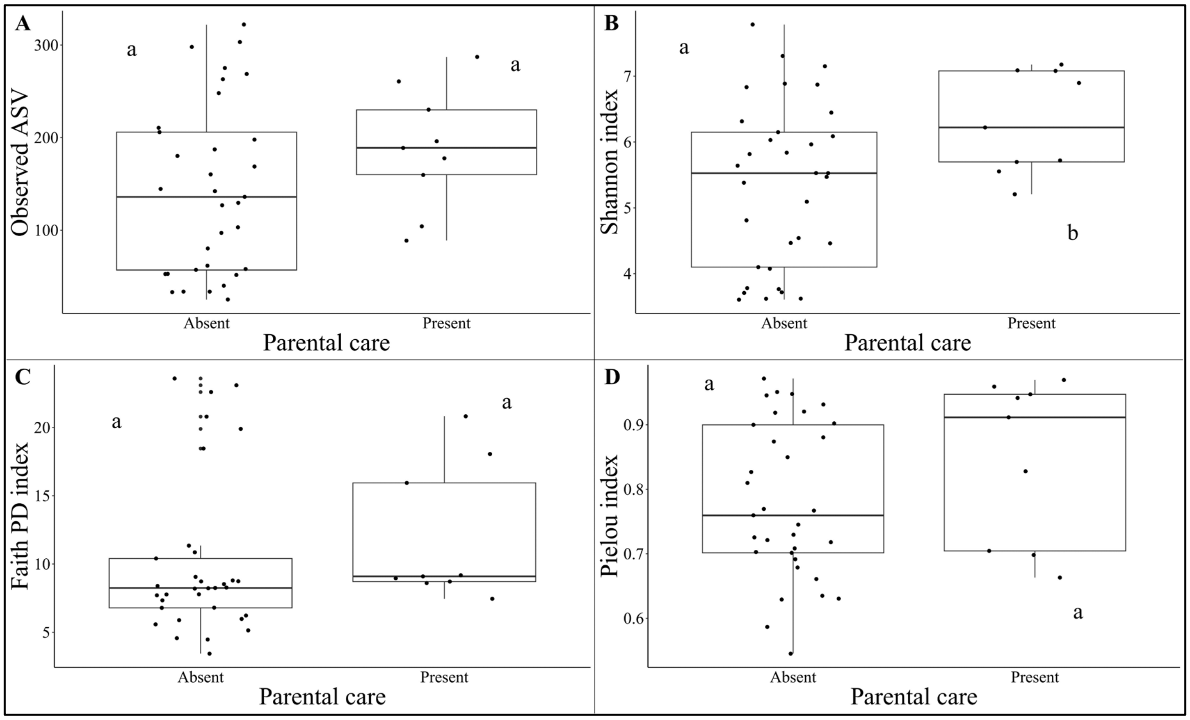

Figure S6. Constrained principal component analysis (CPCA) of intestinal samples based on ASV weighted UNIFRAC matrix. A) tribe, parental care and food preference factors, B) food relocation and habitat factors. Groups are formed by habitat factor

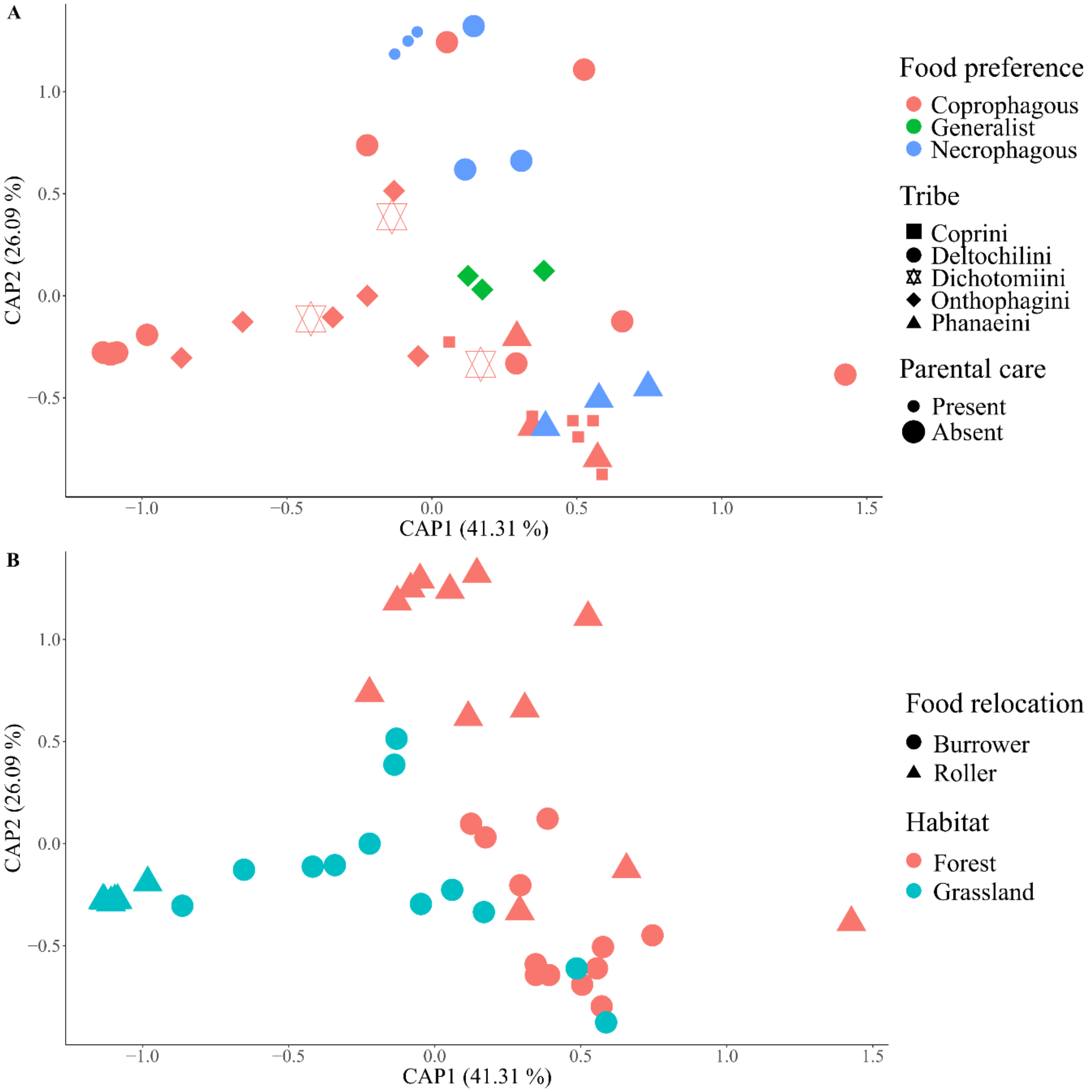

Figure S7. Constrained principal component analysis (CPCA) of intestinal samples based on bacterial genera weighted UNIFRAC matrix. A) tribe, parental care and food preference factors, B) food relocation and habitat factors. Groups are formed by habitat factor.

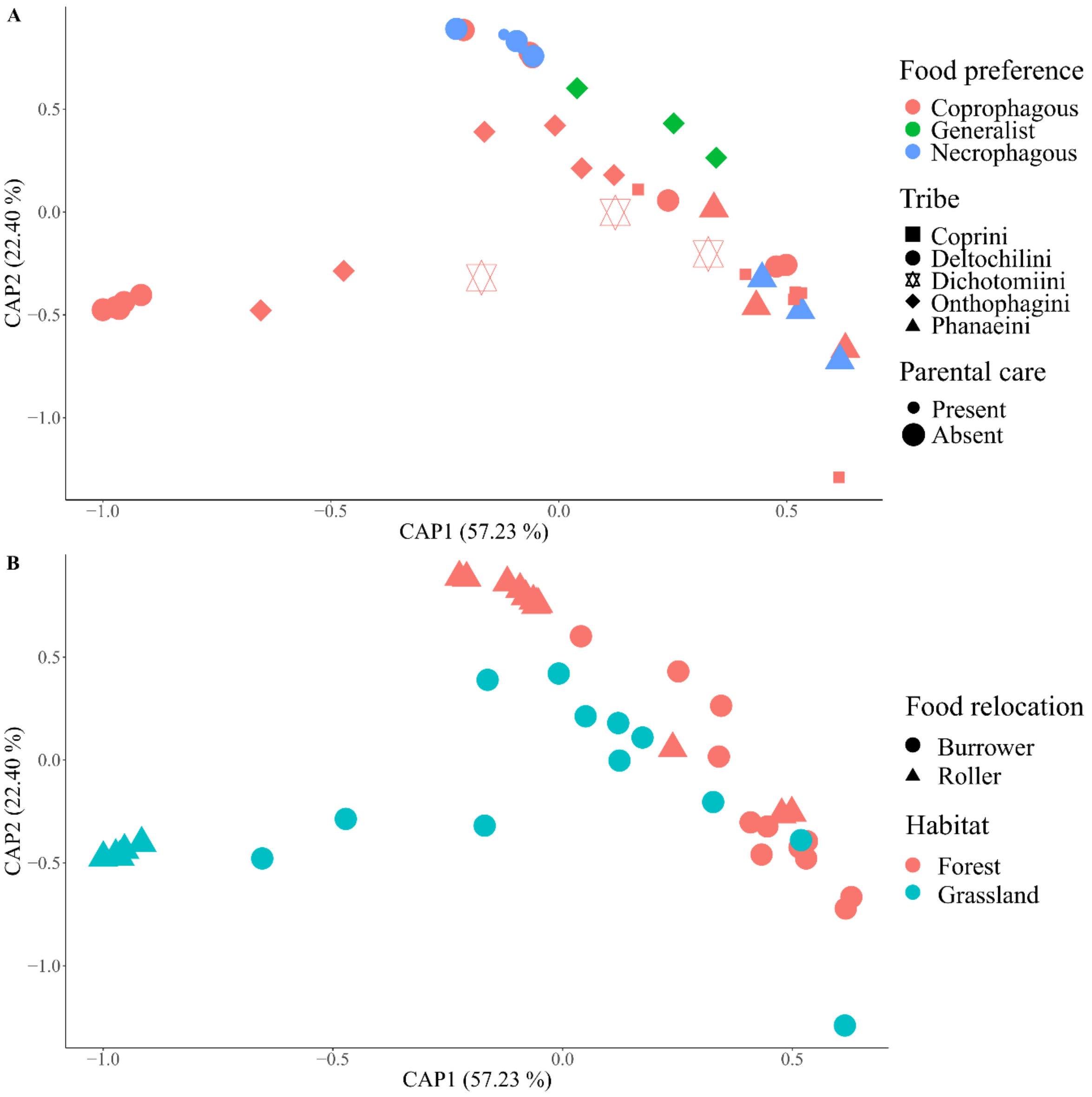

Figure S8. Constrained principal component analysis (CPCA) of intestinal samples based on bacterial genera unweighted UNIFRAC matrix. A) tribe, parental care and food preference factors, B) food relocation and habitat factors. Groups are formed by habitat factor.

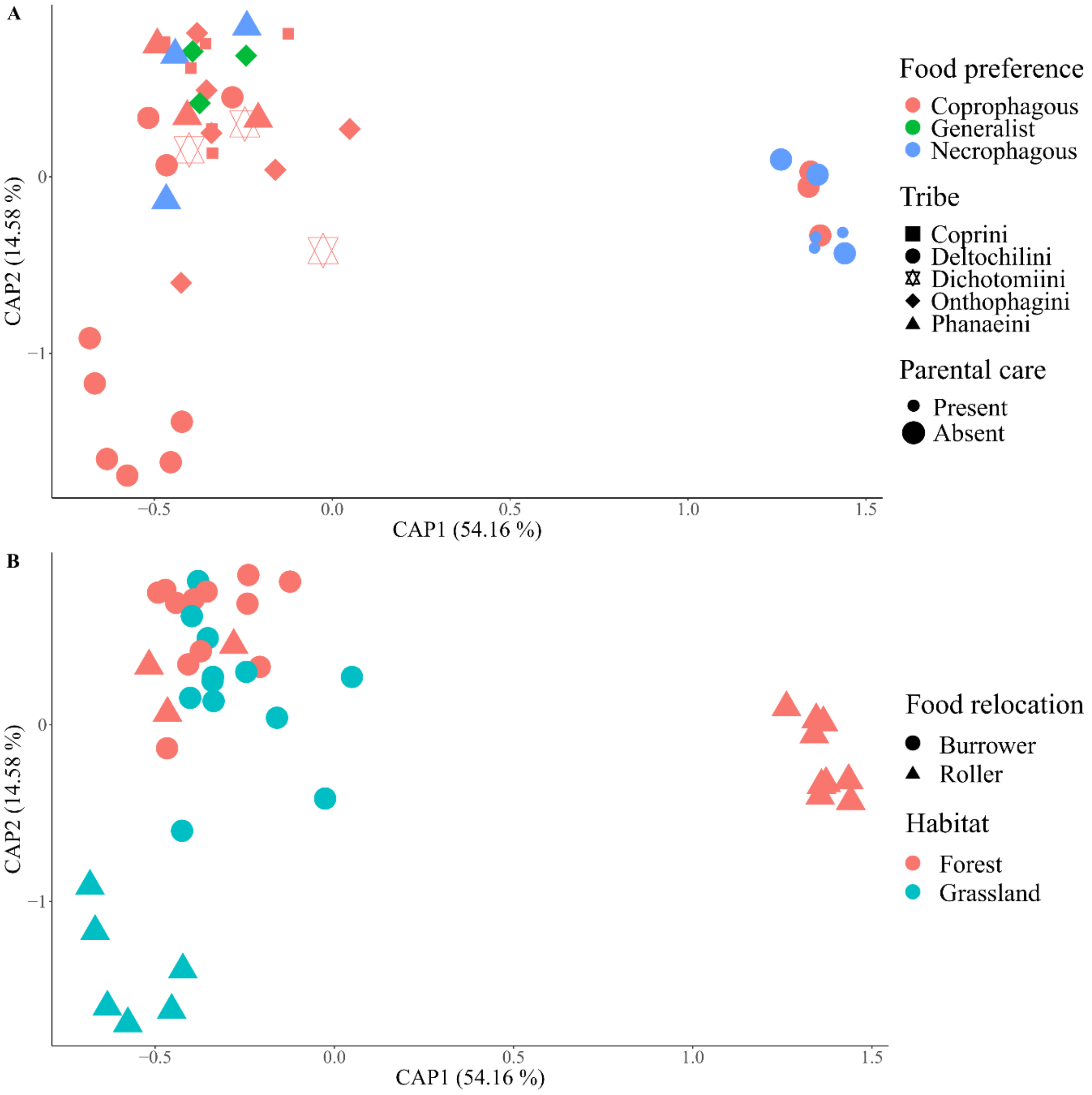

Supplement: Supplementary file 1 [file Data_Sheet_1.pdf]
